# Daily supplementation with lemon verbena extract decreases subjective energy and parental reports of hyperactivity in children displaying sub-clinical attention deficit hyperactivity disorder-type behaviours: A randomised controlled trial

**DOI:** 10.1177/02698811251324574

**Published:** 2025-04-18

**Authors:** Philippa A Jackson, Ellen F Smith, Joanne Forster, Jessica Greener, Anna Small, David O Kennedy, Cynthia G Suarez, Andressa Blainski, Ivo Pischel

**Affiliations:** 1 Brain, Performance and Nutrition Research Centre, Northumbria University, Newcastle upon Tyne, UK; 2 Finzelberg GmbH & Co.KG, Andernach, Germany; 3 Dr. Ivo Pischel Consulting, Rossbach, Germany

**Keywords:** Lemon verbena, *Lippia citriodora*, *Aloysia citriodora*, ADHD, cognition

## Abstract

**Background:**

Current treatment options for attention deficit hyperactivity disorder (ADHD) are limited by factors such as adherence and cost, whilst no treatment options are available for sub-clinical or undiagnosed ADHD. Herbal preparations may therefore offer an alternative approach to the management of symptoms; *Aloysia citriodora* Paláu (lemon verbena) is a promising candidate.

**Aim:**

To assess the behavioural, cognitive, psychological and physiological effects of 56 days of supplementation with lemon verbena extract (LVE) in children exhibiting symptoms of ADHD at the sub-clinical level.

**Methods:**

This exploratory study followed a randomised, double-blind parallel groups design wherein 120 healthy participants aged 8–17 years received 15 mg/kg bw/d LVE or matched placebo for 56 days. Behavioural, cognitive, mood and physiological measures were collected in the lab at baseline and 28 and 56 days post-dose. Parents also evaluated the child’s behaviour throughout the study.

**Results:**

Participants who received LVE reported greater subjective fatigue, defined as reduced energy levels according to the Profile of Mood States subscale, without impairments in cognitive performance across the 56-day intervention and lower depression symptoms on day 56, compared to placebo. The effect of LVE on parent ratings of hyperactive/impulsive behaviour also approached significance with fewer concerns being reported following the active treatment. Exploratory analyses showed further benefits to cognition and mood.

**Conclusions:**

This study revealed novel, beneficial effects of LVE supplementation in children exhibiting a high frequency of behaviours characteristic of ADHD. Overall, LVE was safe and well-tolerated by participants, with no unexpected safety events.

## Introduction

Attention deficit hyperactivity disorder (ADHD) is a chronic neurodevelopmental disorder affecting around 5% of the global population, characterised by a heterogeneous pattern of behaviours including inattention, hyperactivity and impulsivity (Valero et al., 2022). From a neurocognitive perspective, individuals with ADHD have specific challenges in executive functioning, which represents a set of cognitive activities that facilitate decision-making, including working memory, response inhibition, vigilance and planning (Willcutt et al., 2005). ADHD symptoms can persist into adulthood, and the disorder is associated with a greater risk of poor academic and employment outcomes (Erskine et al., 2016). Moreover, two prospective cohort studies concluded that ADHD symptoms in undiagnosed individuals were associated with poorer psychosocial functioning compared to those with a diagnosis (Madsen et al., 2018) as well as healthy controls (Okumura et al., 2021), representing a significant issue for undiagnosed individuals with ADHD symptoms. In the UK, one survey of 10,438 children between the ages of 5 and 15 years found the prevalence of ADHD was 3.62% of boys and 0.85% of girls (Hire et al., 2018), suggesting perhaps an underdiagnosis in this country and therefore inadequate provision of treatment. Treatments for ADHD symptoms include pharmacological or psychosocial interventions, although both have their limitations in terms of side effects and adherence (pharmacological) or cost (psychosocial) (Valero et al., 2022). Alternative treatment options should therefore be considered, particularly for those without access to formal routes of treatment including undiagnosed cases or those who have failed to meet the strict diagnostic criteria. Non-pharmacological natural products such as plant extracts present a potential avenue of alternative treatment for sub-clinical populations. For example, data from our lab revealed that 8 weeks of supplementation with 30 mg/d saffron extract improved sub-clinical depressive symptoms in healthy individuals (Jackson et al., 2021). From another lab, 400 mg/d aqueous extract of lemon balm resulted in significant improvements in mood, mental well-being and quality-of-life scores of participants following 3 weeks of supplementation, who all had moderate depression, anxiety and stress or poor sleep at baseline (Bano et al., 2023). Specific to ADHD symptoms, *Aloysia citriodora* Paláu (lemon verbena) extract could be a promising candidate.

The aromatic shrub lemon verbena – native to South America but also cultivated in North Africa, Southern Europe and Iran – is used throughout the world for medicinal purposes and as a food additive and in drinks. Lemon verbena infusion contains significant amounts of polyphenols, such as phenylpropanoid glycosides, especially verbascoside and flavone diglucuronides such as luteolin-7-diglucuronide, which has high antioxidant activity (Bahramsoltani et al., 2018). Traditionally, lemon verbena has been applied widely due to its antispasmodic, antipyretic, sedative, digestive, antimicrobial and antioxidant properties (Bahramsoltani et al., 2018). Recently, pre-clinical and human studies have provided evidence of sedative and anxiolytic effects. For example, one dose-ranging study in rodents that compared the effects of ethanolic and aqueous extracts of lemon verbena as well as doses of verbascoside showed that all active treatments exhibited anxiolytic, hypnotic and muscle relaxant effects, with exaggerated effects following the highest doses (Razavi et al., 2017). The observed effects were attenuated by the GABA_A_ receptor antagonist flumazenil, suggesting that lemon verbena extract (LVE) and verbascoside specifically may share the same mechanism of action as benzodiazepines, well-known GABA agonists. Additional data from a pilot trial data identified noradrenaline and dopamine reuptake inhibition as a potential mechanism of action (Feistel et al., 2023). Available data on the effects of lemon verbena on behavioural outcomes in humans are currently limited. In one study, 4 weeks of supplementation with 10 cc of lemon verbena essential oil syrup (total essential oil 1.66 mg/10 ml) before bedtime significantly improved sleep quality and insomnia severity in insomnia patients, compared to placebo (Afrasiabian et al., 2019). Similar effects were observed in a recent study in healthy participants currently experiencing moderate levels of stress and subjective poor sleep quality (Martínez-Rodríguez et al., 2022). Following 8 weeks of supplementation with 400 mg purified extract of lemon verbena leaves 1–2 h before bed (standardised to a minimum of 28% total phenylpropanoids), participants reported reduced perceived stress and reduced cortisol in blood along with improved sleep compared to baseline. In comparison to the placebo, the effect of improved sleep quality was only evident in females. Similarly, in healthy adults with sleep disturbances, improvements in sleep quality as assessed by actigraphy and the Pittsburgh Sleep Quality Index were observed following 90-day supplementation with 400 mg LVE (Pérez-Piñero et al., 2024). Furthermore, a dose-finding study in humans with 400, 600 and 800 mg delivered data based on electroencephalogram (EEG) methodology, showing the brain activity, the effective human dose and safety aspects of an LVE (Suarez et al., 2023). Overall, despite limited data, initial findings are promising with regard to the efficacy of lemon verbena as a calming agent.

Given the above, this study aimed to assess the behavioural, cognitive, psychological and physiological effects of LVE and a matched placebo prior to and after 4 and 8 weeks of supplementation in children exhibiting symptoms of ADHD at the sub-clinical level.

## Methods

### Study design

This exploratory study followed a randomised, double-blind, placebo-controlled, parallel groups design. Participants attended the Brain, Performance, Nutrition Research Centre laboratory at Northumbria University, Newcastle upon Tyne, UK, and were assessed after 28 and 56 days of supplementation with LVE, or a matched placebo. Additional online assessments took place on days 14 and 42. The study was performed in accordance with the ethical principles that have their origin in the Declaration of Helsinki from 1996 and commenced only when a favourable ethical opinion was obtained from the Northumbria University Ethical Approval System, UK, approval number 49191. The trial was pre-registered via www.clinicaltrials.gov (NCT05476549).

### Determination of sample size

In the absence of relevant prior data, no primary outcome measure was identified and the power calculation was based on the assumption of an anticipated medium treatment effect size (*f* = 0.25), which indicated that 130 participants (65 per group) would allow detection of significant treatment effects with a power of 0.9 at α = 0.05 in a repeated measures design with two treatment groups and two post-dose time points. Correlation across time was assumed to be *r* = 0.5. For the variables with four post-dose time points, analysis of the same sample size would expect to achieve a power of 0.95. The overall estimate was rounded up to 150 (75 per group), to allow for study dropouts and potential data loss at blind data review.

### Study population

**Figure 1.**
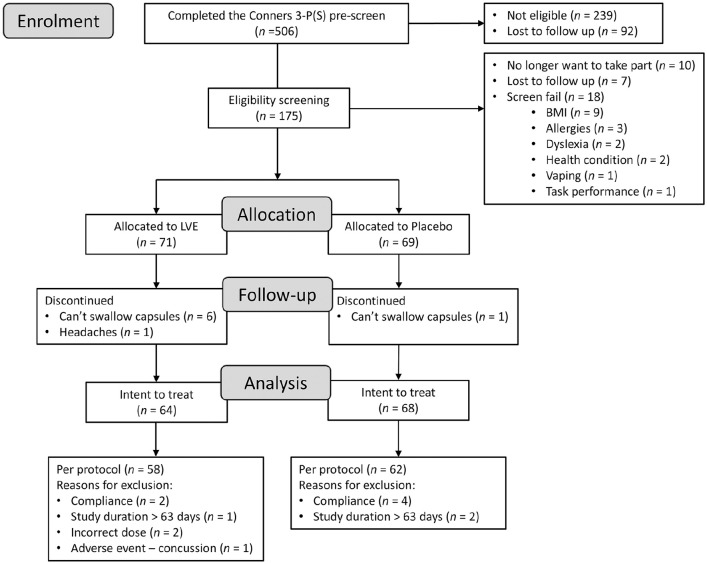
Participant disposition flowchart.

It was intended that 150 participants would be equally stratified into three age groups comprising 8–11, 12–14 and 15–17 year olds. However, towards the end of the enrolment period, it became clear that recruiting 50 participants in the 15–17 years age group would extend the study duration beyond what was acceptable. Therefore, the decision was made to re-open recruitment in the two younger age categories. The end of the UK summer school holidays was chosen as an arbitrary but logical end date for the study; the final participant was enrolled on 25 June 2023 to a total of 140 randomised participants. Eight participants discontinued the study and a further 12 were removed at the blind data review due to major protocol violations leaving a final sample of 120 participants for the analysis. For a full summary of enrolment and the flow of participants, see Figure 1.

Participants were aged between 8 and 17 years, self-reported good health which was confirmed by their guardian, and they scored ⩾60 on the Conners 3-Parent (Short) (Conners 3-P(S)) inattention and hyperactivity/impulsivity (HI) subscales prior to enrolment. They were free from any relevant medical condition or disease including psychiatric and neurodevelopmental disorders. Height and weight data were collected at screening and all participants had a sex- and age-related BMI less than the 98th centile according to the local National Health Service guideline. Participants confirmed that they did not use any prescription medications, alcohol, nicotine or any illicit drugs and were not currently consuming any dietary supplements. They also confirmed that their average daily consumption of caffeine was less than 250 mg/d. A full list of the eligibility criteria can be found in Supplemental File 1. Written, informed assent was obtained from participants, and written informed consent from their guardians prior to any research-related procedures being performed. Participants were recruited via an opportunity sample from Northumbria University students and staff and the wider community using email distribution lists, social media channels and paid newspaper adverts.

### Treatment, randomisation and blinding

Participants received LVE (*Aloysia citriodora* Paláu) manufactured under Good Managing Practice conditions with extract solvent Ethanol 30% V/V. The resulting dry extract is 70% extract, 30% maltodextrin manufactured by Finzelberg GmbH & Co.KG (Andernach, Germany), WO2020099595, branded as MENTALIFY. The placebo treatment was matched for appearance and contained only maltodextrin. Treatments were delivered from the manufacturer identified only by the manufacturer’s code. The randomisation list was computer generated using the online website https://www.randomizer.org, with randomisation codes being generated in 15 blocks of 10, with 5 blocks allocated to each of the 3 age groups. Treatments were labelled by the lab manager, who was also responsible for blinding and creating the emergency code break envelopes. They had no further involvement with the study. All participants, study site personnel apart from the lab manager, and the sponsor representatives involved in the study conduct were kept blinded during the entire study period. No emergency unblinding was needed at any time during the study. The blinded treatment code was only broken after the completion of the main and subgroup statistical analysis; the exploratory analysis was completed after unblinding.

Participants were sequentially allocated to a treatment group according to the randomisation list at the day 1 testing visit with the dose administered based on their body weight. LVE capsules were provided in 200 or 300 mg doses, which were combined to produce the required overall dose of ~15 mg/kg bw/d. Therefore, participants consumed three or four capsules of treatment per day, split between three/four bottles, to account for the differing doses of each type of capsule (200, 300 mg, placebo), depending on their body weight. See Online Supplemental Tables for a table of calculations of dose by body weight. Participants allocated to the placebo group received an equivalent number of capsules of placebo treatment for their weight range.

Participants consumed their daily treatment at home with water before breakfast. They were instructed to consume one capsule from each bottle per day. Due to the size of the capsules (size 0) and the age of the participants, they were permitted to consume the capsules with a little yogurt or a similar vehicle to aid swallowing. They were instructed to completely avoid any product containing grapefruit at breakfast due to contraindications with the active treatment. Participants/guardians completed a daily dosing diary with the time of treatment consumption.

### Behavioural measures

#### The Conners 3 Parent Rating Scale (Short) (Conners 3-P(S))

The Conners 3-P(S) is a popular research and clinical tool for obtaining parental reports of childhood behaviour problems (Conners et al., 2011). The 45 items of the Conners 3-P(S) return six subscales that are analogous to the Conners 3-Self Report (see below): H/I, Inattention, Learning Problems, Defiance/Aggression, Executive Functioning and Peer Relations. Four-point Likert-type scales are used, ranging from 0, not true at all to 3, very much true. Normative data are derived from a large community-based sample of children (US/Canada). Norms are available for children and adolescents aged 6–18 on the parent and teacher rating forms. According to the manual, scores ⩾ 60 for each of the subscales are in the 84th centile and above, which translates from high to very elevated concerns for the behaviours measured by each of the subscales.

#### The Conners 3 Self Reporting Scale (Short) (Conners 3-SR(S))

The Conners 3-SR(S) is a 41-item self-report instrument derived from the Conners 3-SR that is a valid and reliable self-report tool when used with adolescents (Conners et al., 2011). Akin to the Conners 3 parent forms, the Conners 3-SR(S) has five subscales: H/I, Inattention, Learning Problems and Family Relations. Four-point Likert-type scales are used, ranging from 0 to 3 as above. Normative data are derived from a large community-based sample of children (US/Canada). Norms are available for children aged 6–18 on the self-report forms.

### Psychological measures

Visual Analogue Mood Scales (VAMS) – Participants completed 18 visual analogue scales to assess their current mood state, with each anchored by adjectives describing opposite mood states (e.g. Lethargic – Energetic, Tranquil – Agitated, Tense – Relaxed). The data from the individual scales are collapsed into three factors previously identified by factor analysis: ‘alertness’, ‘stress’ and ‘tranquillity’ (see Wightman et al. (2020) for more details).

Perceived Stress Scale (PSS-10) – The PSS-10 is a 10-item questionnaire that assesses the degree to which situations in one’s life are appraised as stressful using a 5-point scale (0–4) with a higher score indicating more perceived stress. It is a widely used research instrument and its validity has been established within a number of populations (Golden-Kreutz et al., 2004; Mimura and Griffiths, 2004).

Profile of Mood States 2 Youth Form (POMS 2-Y) (McNair et al., 1992) – The POMS is a well-established, factor-analytically derived measure of psychological distress for which high levels of reliability and validity have been documented in 13–17 year olds. The POMS 2-Y Short form consists of 35 adjectives rated on a 0–4 scale that can be consolidated into six subscales: depression–dejection, tension–anxiety, anger–hostility, confusion–bewilderment, vigour–activity and fatigue–inertia. The latter two subscales can be interpreted as measures of fatigue and have been validated as separate factors in a number of studies.

State-Trait Anxiety Inventory (STAI) (Spielberger et al., 1969) – The STAI ‘State’ subscale is a widely used instrument for measuring fluctuating levels of anxiety. The subscale contains 20 statements (e.g. ‘I am calm’) each with a 4-point Likert scale. Participants rate how much they feel about each statement at the time of making the response. Scores on the STAI range from 20 to 80, with higher scores representing greater levels of anxiety.

### Cognitive measures

Cognitive function was assessed using the Computerised Mental Performance Assessment System (COMPASS) (Northumbria University). This testing system delivers a bespoke collection of tasks, with fully randomised parallel versions of each task delivered at each assessment for every participant. It has previously been shown to be sensitive to a wide range of nutritional interventions (Kennedy et al., 2011, 2017a, 2017b; Stonehouse et al., 2013). The commercially available battery has been in use within our laboratory for over 15 years and is currently in use within a number of UK, US, New Zealand and Australian Universities and research organisations.

Meta-analyses suggest that children with ADHD exhibit deficiencies in executive function, working memory and selective attention. Specific tasks sensitive to the condition include response inhibition tasks (e.g. Stroop task (Homack and Riccio, 2004)), selective/focussed/sustained attention tasks and verbal/spatial working memory tasks (Homack and Riccio, 2004; Kasper et al., 2012; Martinussen et al., 2005; Ramos et al., 2020; Willcutt et al., 2005). Therefore, the selection of tasks employed here comprised several standard and ‘classic’ tasks that assessed aspects of memory (working, spatial), cognitive flexibility, attention (selective, focussed, sustained) and executive function. These were the arrow flankers task, rapid visual information processing, the Stroop task, computerised Corsi blocks task, numeric working memory and peg and ball task. One potential advantage of the COMPASS battery is the possibility of collapsing the task outcomes into ‘factors’ which can be useful to establish if the treatment has a global effect on a given cognitive domain that might escape significance on the component tasks. Two global scores were calculated: Speed of Performance – comprising reaction time (msec) data from all tasks that return these data; and Accuracy of Performance – comprising % accuracy data from all of the tasks that return these data. These tasks and outcomes are described in detail in Supplemental File 2.

### Physiological measures

Sitting blood pressure data were collected using a Boso Medicus Prestige blood pressure monitor with the participant’s arm supported at the level of the heart and with their feet flat on the floor. Readings were taken following a 5-min rest during the pre-assessment interview at each testing visit. Temperature was measured at the same time as blood pressure using MediPro non-contact infrared thermometer. Heart rate data were collected continuously throughout the entire testing visit using the Polar H10 heart rate monitor and connected via Bluetooth to a Polar Unite watch (Polar Electro, UK) that were applied following the blood pressure measurement. Data were subsequently exported from the watch using the Polar FlowSync software (Polar Electro, UK) and transformed into heart rate variability (HRV) data using Kubios HRV Scientific 4.0.1 (Kubios Oy, Finland). Using this software, two samples from the data were derived to reflect HRV at rest (a 2-min sample during the completion of the questionnaires) and during the performance of cognitive tasks (a 5-min sample during the completion of the tasks).

### Procedure

Recruitment and erollment took place between August 2022 and June 2023. All data were collected at the Brain, Performance and Nutrition Research Centre, Northumbria University, Newcastle upon Tyne, UK. Following recruitment and the distribution of participant information sheets, an initial pre-screening questionnaire comprising the Conners 3-P(S) was completed. Individuals who met the Conners 3-P(S) eligibility criterion proceeded to have a remote screening session followed by visits to the laboratory on four separate occasions: an introductory/training visit and three active testing visits (Day -1, Day 28 and Day 56).

The remote screening session was completed via video/telephone call and comprised: briefing on the requirements of the study, obtaining of informed assent (child) and consent (parent/guardian) via completion of an online consent form, health screening, collection of demographic data and completion of a caffeine consumption questionnaire. The introductory/training visit to the laboratory began with physiological eligibility measures that could not be completed remotely (i.e. height and weight), followed by training on the completion of the cognitive and psychological/mood measures.

Following the introductory/training visit, participants attended the laboratory in the morning on three separate occasions (Day -1, Day 28, Day 56 with respect to treatment administration). All laboratory testing visits took place at the weekend and each of these laboratory assessments was identical, comprising collection of blood pressure, temperature and heart rate measurements, the completion of behaviour (Conners 3-SR(S)) and psychological state (STAI, POMS, PSS, VAMS) questionnaires and the COMPASS computerised cognitive assessment. Participants completed the questionnaires and cognitive assessments in temperature-controlled labs with participants visually isolated from each other and were also instructed to wear noise cancelling headphones to aid concentration. The cognitive assessment took approximately 25 min to complete. At the end of the laboratory visits on Day -1 and 28, the participants received the treatment that they were instructed to consume daily prior to the next visit, starting on the following day. At the end of Day 56, the participant’s treatment compliance was checked via capsule count verified by the diary.

**Figure 2.**
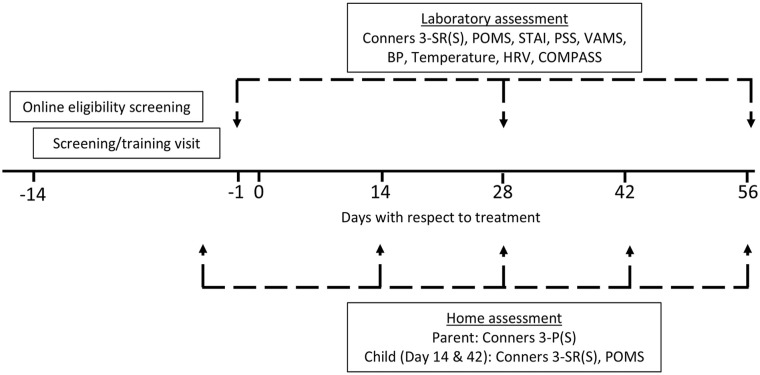
Timeline of the study assessment schedule. Having successfully passed the online pre-screen Conners 3-P(S) questionnaire, participants attended their screening/training visit between 14 and 2 days before treatment commenced. They then attended their full testing visits (comprising laboratory assessments of self-reported mood/behaviour and cognitive function) the day before (Day 1), 28 days (Day 28) and 56 days (Day 56) after treatment commenced. A concomitant assessment of parents’ perceptions of their child’s behaviour took place prior to Days 1, 14, 28, 42 and 56. On Days 14 and 28, participants also completed the Conners 3-SR(S) and POMS at home. Conners 3-SR(S): conners 3-self report form (short); Conners 3-P(S); conners 3-parent form (short); POMS: profile of mood states; STAI: state-trait anxiety inventory; PSS: perceived stress scale; VAMS: visual analogue mood scales; BP: blood pressure; HRV: heart rate variability; COMPASS: cognitive testing platform.

Parents and participants also took part in at-home assessments that were completed online, comprising the collection of the parent’s assessment of the child’s behaviour/cognitive function (Conners 3-P(S)) and the child’s self-report of the same, plus their psychological state (Conners 3 SR(S) and POMS). For the parents, these assessments took place on Day -1, 14, 28, 42 and 56. For the participants, home assessments took place on Days 14 and 42. The timeline of the whole study including the home assessments is shown in Figure 2.

### Statistical analysis

Data collected on Day -1 were analysed via *t*-tests to ascertain whether any differences between groups were present at baseline. The MIXED procedure in SPSS version 28.0.1.1 (IBM corp., Armonk, New York, USA) was used to analyse all outcomes. Treatment (LVE, placebo) and day (14, 28, 42 and 56, as appropriate) were entered into the model as fixed effects along with their interaction (treatment × day) and respective baseline (Day -1) score as a covariate. Age (in months) was entered into the model, if it improved model fit, as determined by Schwartz’s Bayesian Criteria, which was the case for all but the Conners 3-SR(S) and Conners 3-P(S) outcome measures. The first-order autoregressive covariance structure of the residuals (AR1) was selected as the most appropriate for the repeated measures term (day) in all models. Significant interaction effects were analysed further using pairwise comparisons with the least significant difference adjustment, given the exploratory nature of the investigation. Missing data were assumed to be missing at random. Statistical significance was set at *p* < 0.05 for the main analysis and trends between 0.05 < *p* < 0.1 were considered for the exploratory analysis. Given the previously reported age and sex differences in ADHD (Gilbert et al., 2023), planned subgroup analyses running the above models for all outcomes with data split by sex (male, female) and age (8–11, 12–14, 15–17 years) were performed, the descriptive data and results of which are presented in the Online Supplemental Tables – Subgroup analysis only, for brevity.

## Results

The flow of participants through the study is summarised in Figure 1. The final analysis was conducted in 120 participants (*n* = 58 in the LVE group; *n* = 62 in the placebo group) for whom baseline and end of study data were available and who did not meet any major protocol violations (compliance, neurological adverse event unrelated to treatment). Baseline characteristics of participants are summarised in Table 1.

**Table 1.** Demographic and baseline characteristics of participants. *p*-values were derived from independent samples *t*-test for continuous variables and Chi-squared test for categorical variables.

	LVE (*N* = 58)		PLA (*N* = 62)		*p*
Baseline characteristic	Mean	SD	Mean	SD	
Age (years)	12.00	2.35	12.29	2.72	0.532
Weight (kg)	49.51	13.35	47.48	13.03	0.401
Height (m)	157.23	13.58	155.30	14.25	0.449
Body mass index (kg/m^2^) percentile	59.22	30.34	60.63	31.19	0.402
Caffeine consumption (mg/day)	17.51	35.48	16.20	30.67	0.830
Fruits and vegetables consumption (portion/day)	3.37	1.43	3.54	1.44	0.519
	*N*	%	*N*	%	*p*
Sex (as assigned at birth)					
Male	29	50.0	39	62.9	0.154
Female	29	50.0	23	37.1	
Ethnicity					
White	50	86.2	54	87.1	0.936
Asian (Indian, Bangladeshi, Pakistani)	3	5.2	2	3.2	
Chinese	3	5.2	3	4.8	
Other	2	3.4	3	4.8	
Use of glasses					
Yes	9	15.5	21	33.9	0.020
No	49	84.5	41	66.1	
Handedness					
Left	6	10.3	7	11.3	0.868
Right	52	89.7	55	88.7	
Dietary habits					
No dietary restrictions that is, eating meat	50	86.2	58	93.5	0.347
Vegetarian	4	6.9	1	1.6	
Pescatarian	1	1.7	1	1.6	
Vegan	3	3.0	2	3.2	

### Baseline comparisons

Several significant between-group differences were identified for the following outcome measures: Conners 3-P(S) HI (*p* = 0.019), learning problems (*p* = 0.012) and executive functioning (*p* = 0.018) and defiance/aggression (*p* = 0.044), POMS depression-dejection (*p* = 0.025) and tension-anxiety (0.027), PSS score (*p* = 0.040) and STAI score (*p* = 0.028). Across all these measures, participants randomised to receive LVE had higher scores compared to placebo. Data and analyses are listed in the Online Supplemental Tables – Main analysis.

### Compliance and treatment guessing

Although average compliance was high in both groups, participants who received LVE had lower compliance (94.5% ± 7.95) compared to those who received placebo (98.3% ± 6.1), (*t*(117) = 2.952, *p* = 0.004). Both participants and their parents were invited to guess the treatment that they/their child had received during the study. Chi-squared analysis revealed that there was no significant difference between treatment groups as rated by the parents (*p* = 0.260) or children (*p* = 0.140), confirming the success of the blinding procedure. However, interestingly, 63% of participants who correctly guessed that they had received LVE (*n* = 24) cited reasons related to beneficial effects including a calming effect, improved concentration/focus, improved mood and improved school performance. The remaining 26% thought they had received LVE because of taste/appearance or were under the impression that everyone in the study received the active treatment. Of the 28 participants who incorrectly guessed they had received LVE, only 50% cited positive effects, and the other half cited taste/appearance believed everyone in the study received LVE, felt sleepier or gave no reason.

### Main analysis

**Figure 3.**
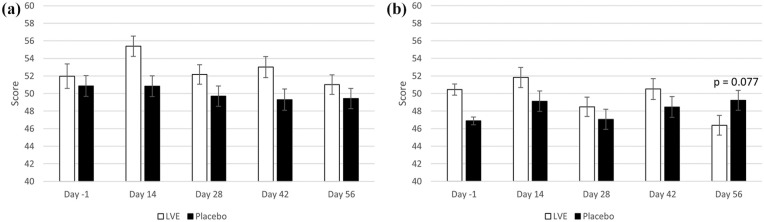
POMS subscales. Scores are separated by day and treatment. Day -1 data are raw means. Post-dose data are estimated marginal means derived from the linear mixed model that is adjusted for baseline score. (a) Fatigue-Inertia – A significant main effect of treatment was observed (*F*(1, 153.29) = 12.79, *p* < 0.001). (b) Depression-Dejection – pairwise comparisons following a significant treatment × day interaction revealed a trend for lower depression on Day 56.

A significant effect of treatment was detected for the fatigue–inertia subscale of the POMS (*F*(1, 141.45) = 11.54, *p* = 0.001). Participants who received LVE reported significantly more fatigue (*M* = 52.90, SEM = 0.63) compared to placebo (*M* = 49.8, SEM = 0.64) during the study. In addition, a significant treatment × day interaction was observed for the depression-dejection subscale (*F*(3, 286.94) = 2.72, *p* = 0.045). Pairwise comparisons revealed a trend for lower depression on Day 56 following LVE (*M* = 46.38, SEM = 1.12) compared to placebo (*M* = 49.22, SEM = 1.13) (*p* = 0.077). See Figure 3.

**Figure 4.**
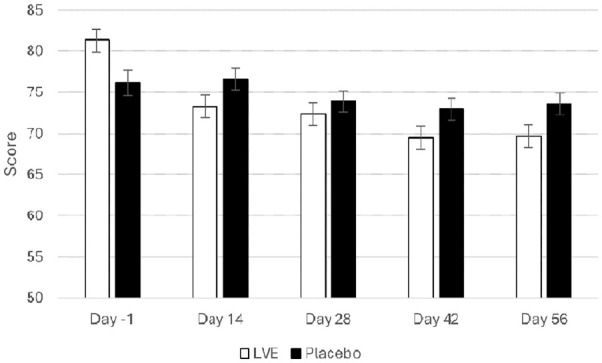
Conners 3-P(S) hyperactivity/impulsivity scores separated by day and treatment. Day -1 data are raw means. Post-dose data are estimated marginal means derived from the linear mixed model that is adjusted for baseline score. A near-significant effect of treatment was observed (*F*(1, 125.67) = 3.84, *p* = 0.052).

A main effect of treatment approached significance for the Conners 3-P(S) HI subscale (*F*(1, 125.67) = 3.84, *p* = 0.052), where the LVE group’s parents reported fewer concerns for these behaviours (*M* = 71.22, SEM = 1.11), compared to those of participants who received placebo (*M* = 74.28, SEM = 1.10). See Figure 4.

Descriptive data and analysis of all outcomes in the main analysis are presented in the Online Supplemental Tables – Main analysis.

### Exploratory analysis

**Figure 5.**
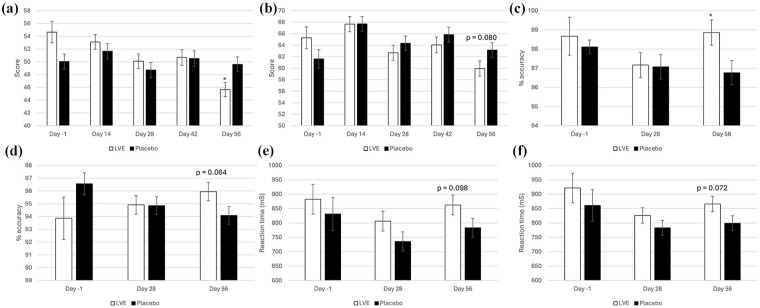
Exploratory analysis of Day 56 treatment effects. Day -1 data are raw means. Post-dose data are estimated marginal means derived from the linear mixed model that is adjusted for baseline score; significance is derived from pairwise comparisons on Day 56. (a) POMS Tension-Anxiety; (b) Conners 3-SR hyperactivity/impulsivity; (c) Stroop task congruent stimuli accuracy; (d) overall Stroop task accuracy; (e) arrow flankers task congruent stimuli reaction time; (f) arrow flankers task overall reaction time. *p < 0.05.

In the process of performing the main and subgroup analyses (age, sex), the authors noted a pattern of differences between the treatments at Day 56, with pairwise comparisons revealing several beneficial effects of LVE worthy of note for future investigation. These were reduced POMS Tension-Anxiety (LVE *M* = 45.66, SEM = 1.14; placebo *M* = 49.61, SEM = 1.16; *p* = 0.016), reduced Conners 3-P(S) HI (LVE *M* = 69.73, SEM = 1.36; placebo *M* = 73.55, SEM = 1.31; *p* = 0.045) and a trend in the same direction for Conners 3-SR HI (LVE *M* = 59.93, SEM = 1.33; placebo *M* = 63.16, SEM = 1.27; *p* = 0.080), improved accuracy on the Stroop task congruent stimuli accuracy (LVE *M* = 98.86, SEM = 0.66; placebo *M* = 96.77, SEM = 0.63; *p* = 0.023) and a trend in same direction for overall Stroop task accuracy (LVE *M* = 95.96, SEM = 0.72; placebo *M* = 94.09, SEM = 0.69; *p* = 0.064). Conversely, a trend for slower reaction time to the congruent stimuli on the arrow flankers task following LVE at Day 56 was observed (LVE *M* = 862.60, SEM = 34.42; placebo *M* = 783.31, SEM = 33.03; *p* = 0.098), with a trend in the same direction detected for overall reaction time on this task (LVE *M* = 865.98, SEM = 26.67; placebo *M* = 798.95, SEM = 25.62; *p* = 0.072). See Figure 5. See Figure 4 for Conners 3-P(S) HI.

With regard to safety, 131 individual adverse events were reported, with a similar number of adverse events being reported in each treatment group (71 vs 60 following LVE and placebo respectively). Adverse events were not related to treatment and included reporting of colds, headaches, aches and pains, gastrointestinal upset, etc. Therefore, the treatment was safe and well-tolerated by participants, with no unexpected safety events.

## Discussion

To our knowledge, the current exploratory study is the first to investigate the effects of a herbal supplement derived from Lemon Verbena on the behaviour, cognition and mood of children who exhibited a high frequency of inattentive and hyperactive/impulsive (H/I) type behaviours at enrolment as rated by parents, but who do not have a diagnosis of ADHD. According to the Conners 3 manual (Conners et al., 2011), common characteristics of children who score highly on the inattention subscale include poor concentration/attention or difficulty keeping their mind on work, may make careless mistakes, may be easily distracted, may give up easily or is easily bored, may avoid schoolwork and may have difficulty starting and/or finishing tasks. For the H/I subscale, children who score highly here have high activity levels, may be restless and/or impulsive, may have difficulty being quiet, may interrupt others or talk too much and may be easily excited. At baseline, the mean Conners 3-P(S) score for the entire sample for inattention was 76.81 (±12.72) and for H/I was 78.71 (±11.71), indicating that the level of concerns of parents regarding the types of behaviour captured by these measures was in the top 2% of standardised scores. At the same time point, children’s rating of their behaviour on inattention and H/I using the Conners 3-SR was also higher than average, although not as high as parent ratings: 65.05 (±14.61) and 63.48 (±13.36), respectively. The study intended to recruit participants with sub-clinical ADHD symptoms; given the average sample scores on inattention and H/I, it can be concluded that in this respect, the study was successful.

The main analysis revealed that participants who received LVE reported reduced energy levels, as measured by the POMS fatigue subscale, across the 56-day intervention, along with lower depression symptoms by Day 56 compared to placebo. The effect of LVE on parent ratings of H/I behaviour also approached significance, with fewer concerns being reported following the active treatment. The exploratory analysis of Day 56 data revealed further beneficial effects on mood (anxiety), H/I behaviours reported by both parents and children and cognition (Stroop task accuracy). To be clear, while no primary outcome measure was indicated at the study outset, it should be noted that none of the key indicators of ADHD symptoms – parent and self-reported inattention and hyperactivity – reached statistical significance following LVE compared to placebo. However, a notable trend towards improvement was observed on several measures, suggesting potential benefits that warrant further investigation.

LVE was selected as an intervention in this specific population due to its previously reported calming and sedative effects in traditional medicine (Bahramsoltani et al., 2018), rodent models (Ragone et al., 2010; Sabti et al., 2019) and humans (Afrasiabian et al., 2019; Martínez-Rodríguez et al., 2022). Our findings suggest that the putative sedative effects of LVE were experienced and/or reported as fatigue. However, although fatigue usually has some negative connotations, it may be the case that for this sample of participants, a reduction in energy is in fact a novel beneficial effect of LVE. This suggestion is also supported by the observed decrease in H/I concerns reported by parents (main analysis) and the participants themselves (exploratory analysis). It is useful to note here that one recent acute dose-ranging crossover study in a small sample of females (*N* = 10, healthy, mean age 55 years) revealed a trend for increased fatigue as measured using the POMS following 600 and 800 mg of the same LVE used in the current study (Suarez et al., 2023, unpublished data). This effect was observed 90 min post-dose which is analogous to the time frame of testing in the current study, as participants took their daily dose at home with breakfast prior to the testing visit. Taking the data from both studies together suggests that the effects of LVE on fatigue are evident from the first dose and do not diminish over time. Importantly, despite the observed increase in self-reported fatigue, it should be noted that this effect was not linked to any consistent decrements in performance on the cognitive tasks. In the future, it would be interesting to include measures specifically indicating positive and negative affect, to better understand how ‘fatigue’ is experienced in this population, and whether the fatigue is felt at the physical and/or mental level.

We also observed evidence supporting the previously observed anxiolytic (Martínez-Rodríguez et al., 2022; Razavi et al., 2017) and anti-depressant (Sabti et al., 2019) effects of LVE in both humans and rodents. Here, the pattern of data for both outcomes indicated a gradual decline in symptoms over the course of the intervention, with the greatest treatment effects observed at the final assessment on Day 56. Taking our data together with previous reports, a consistent pattern of positive effects of this herbal extract on psychological mood state is evident and may indicate LVE in the treatment of common co-occurring symptoms of ADHD that standard pharmaceutical interventions do not address such as emotional dysregulation, depression, anxiety and neurocognitive dysfunction (Gonda et al., 2024). To this end, future studies could also assess the effects of LVE on sleep quality.

One potential mechanism underpinning the observed sedative, anxiolytic and behavioural effects could be the previously reported ability of LVE constituents to bind at GABA_A_ receptors, inducing benzodiazepine-like effects. Candidates here are limonene, citral, luteolin-7-diglucuronide and verbascoside, although a combination of these and the many other terpenoid and polyphenolic compounds comprising the complex chemical profile of LVE may be responsible (Ragone et al., 2010). In addition, Sabti et al. (2019) observed modulation of multiple neurotransmitter systems including serotonin, dopamine and noradrenaline following LVE in a rodent model of induced depression, as well as increased brain-derived neurotrophic factor, all of which may be associated with the effects described above. Indeed, effects on dopamine and noradrenaline reuptake following LVE were also observed in humans (Suarez et al., 2023). The binding of LVE-derived compounds at GABA receptor sites is also particularly relevant given that overall GABA concentration in the brain was found to be lower in children 8–12 years diagnosed with ADHD compared to age-matched controls (Edden et al., 2012). In addition, GABA concentration in the dorsolateral prefrontal cortex was shown to be inversely related to impulsivity in healthy young males (Boy et al., 2011). It is therefore possible that this pharmacological action of LVE may specifically benefit the dopaminergic and GABAergic deficit associated with ADHD-type behaviours and requires further investigation. Of course, it must be considered that the effects observed here are general effects of LVE not specific to ADHD-type behaviours. Therefore, the extent to which our results are replicated in other populations would be of interest, as well as exploring LVE supplementation in individuals experiencing depression, anxiety and stress, for example.

The cognitive effects revealed in the exploratory analysis are more difficult to interpret, which indicates better performance on the Stroop task on Day 56 in terms of accuracy, but slower performance on the arrow flankers task. Both findings may indicate a reduction in impulsive responses following LVE which would link to the above behavioural effects. However, given the exploratory nature of these analyses, caution should be exercised in their interpretation. As above, with regard to the cognitive performance measures, the most important finding to note is that no consistent decrements in performance were observed. Indeed, participants who received LVE reported being able to focus or concentrate better and perform better at school. It would therefore be interesting to consider teacher reports of behaviour, which is also possible using the Conners 3. The subgroup analyses presented in the supplementary materials largely follow the main analysis, but a few interesting differences were noted. For example, the fatigue effect of the treatment was more pronounced in boys, and the effect on depression on Day 56 was only evident in girls. These data suggest that the gender differences observed in the presentation of ADHD symptoms (Gilbert et al., 2023) extend to how they respond to the supplement as well and are worthy of further investigation.

As previously mentioned, this study achieved its aim of recruiting participants with sub-clinical levels of ADHD-type behaviours. In addition, participants included in the analysis were highly compliant with the treatment regimen, and therefore the reported effects can be attributed to a treatment dose of ~15 mg/kg/d for 56 days. However, the study has some limitations. First, it was intended that 150 participants would be recruited, with an even split across all three age categories. Recruitment in the older age group was especially challenging, and when it became apparent that the intended sample size for this group would not be achieved, recruitment in the lower age categories was re-opened. Recruitment delays also meant that the intended sample size of 150 participants was not achieved; following cleaning only 120 out of an anticipated 130 datasets were analysed, suggesting that the study was underpowered particularly with regard to the cognitive measures.

It is unfortunate that several significant baseline group differences were present in the current sample. These differences were in relation to mood (depression–dejection, tension–anxiety, PSS score, STAI score) and behaviour (family problems, learning problems, executive functioning), with participants allocated to the LVE treatment group scoring significantly higher (worse) on all these measures. Although baseline score was included in all analyses as a covariate – meaning all analyses of post-intervention data were adjusted for baseline score – this does not resolve the possibility that the treatment groups were potentially phenotypically different, which may have affected response to treatment. Although double-blind randomisation is the gold standard for ensuring that such a low-probability event does not occur, consideration of this fact does result in a more cautious interpretation of the findings reported herein.

Finally, it was an oversight that a measure of parent socio-economic status was not collected as part of the study, to be better able to characterise the sample of participants that were recruited. However, the mean daily consumption of fruits and vegetables in the sample, which was almost 3.5 portions, is higher than the national average (NHS Digital, 2019), suggesting that our sample of participants was drawn from a more affluent population, and should be taken into consideration when interpreting the data.

In conclusion, 56 days of supplementation with ~15 mg/kg/d LVE in children exhibiting sub-clinical levels of ADHD-type behaviour was associated with higher subjective fatigue across the treatment period and reduced symptoms of depression on Day 56. A near-significant treatment effect also indicated that parents of children who received LVE reported fewer concerns for hyperactive/impulsive behaviours. These findings are in line with the previously reported sedative effects of this herbal preparation. However, despite this, LVE was associated with a consistent pattern of stable cognitive performance and could indicate a beneficial calming or ‘de-energising’ effect in this population. The exploratory analysis of Day 56 data revealed further beneficial effects on cognition, mood and behaviour, compared to a placebo. While the observed effects of LVE may not be entirely specific to ADHD-type behaviours, our findings highlight a pronounced benefit in this population, as demonstrated by changes in fatigue and improvements in mood in the absence of cognitive decrements – a common limitation observed in general applications of aqueous LVEs. Notably, participants in our study showed improved performance on the Stroop test, indicating enhanced selective attention and cognitive flexibility, underscoring the extract’s potential for targeted support in ADHD-related challenges and beyond. Further research across broader populations would help delineate the scope of these effects, in addition to adequately powered follow-up investigations to confirm our initial findings. Overall, the treatment was safe and well-tolerated by participants, with no unexpected safety events.

## Supplemental Material

sj-docx-1-jop-10.1177_02698811251324574 – Supplemental material for Daily supplementation with lemon verbena extract decreases subjective energy and parental reports of hyperactivity in children displaying sub-clinical attention deficit hyperactivity disorder-type behaviours: A randomised controlled trial Supplemental material, sj-docx-1-jop-10.1177_02698811251324574 for Daily supplementation with lemon verbena extract decreases subjective energy and parental reports of hyperactivity in children displaying sub-clinical attention deficit hyperactivity disorder-type behaviours: A randomised controlled trial by Philippa A Jackson, Ellen F Smith, Joanne Forster, Jessica Greener, Anna Small, David O Kennedy, Cynthia G Suarez, Andressa Blainski and Ivo Pischel in Journal of Psychopharmacology

sj-docx-2-jop-10.1177_02698811251324574 – Supplemental material for Daily supplementation with lemon verbena extract decreases subjective energy and parental reports of hyperactivity in children displaying sub-clinical attention deficit hyperactivity disorder-type behaviours: A randomised controlled trial Supplemental material, sj-docx-2-jop-10.1177_02698811251324574 for Daily supplementation with lemon verbena extract decreases subjective energy and parental reports of hyperactivity in children displaying sub-clinical attention deficit hyperactivity disorder-type behaviours: A randomised controlled trial by Philippa A Jackson, Ellen F Smith, Joanne Forster, Jessica Greener, Anna Small, David O Kennedy, Cynthia G Suarez, Andressa Blainski and Ivo Pischel in Journal of Psychopharmacology

sj-xlsx-3-jop-10.1177_02698811251324574 – Supplemental material for Daily supplementation with lemon verbena extract decreases subjective energy and parental reports of hyperactivity in children displaying sub-clinical attention deficit hyperactivity disorder-type behaviours: A randomised controlled trial Supplemental material, sj-xlsx-3-jop-10.1177_02698811251324574 for Daily supplementation with lemon verbena extract decreases subjective energy and parental reports of hyperactivity in children displaying sub-clinical attention deficit hyperactivity disorder-type behaviours: A randomised controlled trial by Philippa A Jackson, Ellen F Smith, Joanne Forster, Jessica Greener, Anna Small, David O Kennedy, Cynthia G Suarez, Andressa Blainski and Ivo Pischel in Journal of Psychopharmacology

sj-xlsx-4-jop-10.1177_02698811251324574 – Supplemental material for Daily supplementation with lemon verbena extract decreases subjective energy and parental reports of hyperactivity in children displaying sub-clinical attention deficit hyperactivity disorder-type behaviours: A randomised controlled trial Supplemental material, sj-xlsx-4-jop-10.1177_02698811251324574 for Daily supplementation with lemon verbena extract decreases subjective energy and parental reports of hyperactivity in children displaying sub-clinical attention deficit hyperactivity disorder-type behaviours: A randomised controlled trial by Philippa A Jackson, Ellen F Smith, Joanne Forster, Jessica Greener, Anna Small, David O Kennedy, Cynthia G Suarez, Andressa Blainski and Ivo Pischel in Journal of Psychopharmacology
