# Supplementary material for: Daily supplementation with lemon verbena extract decreases subjective energy and parental reports of hyperactivity in children displaying sub-clinical attention deficit hyperactivity disorder-type behaviours: A randomised controlled trial
Source: J Psychopharmacol. 2025 Apr 18;39(8):825–35. doi: 10.1177/02698811251324574 (PMC12287557; doi:10.1177/02698811251324574)
Supplement: sj-docx-1-jop-10.1177_02698811251324574 – Supplemental material for Daily supplementation with lemon verbena extract decreases subjective energy and parental reports of hyperactivity in children displaying sub-clinical attention deficit hyperactivity disorder-type behaviours: A randomised controlled [file sj-docx-1-jop-10.1177_02698811251324574.docx]

## Cognitive measures

### Arrows Flankers (selective attention, cognitive flexibility)

Five symbols appeared on the screen with the centre symbol always being an arrow pointing to the left or right. The participant pressed the left or right response box button corresponding to the direction of the central arrow. The flanking pairs of symbols were either congruent arrows (pointing in the same direction), incongruent arrows (pointing in the opposite direction, or squares (neutral stimuli). A total of 90 stimuli, evenly split between congruent, incongruent, and neutral stimuli were presented with a randomly varying inter-stimulus interval of between 1 and 3 seconds. Each stimulus remained on screen until the response was registered. Outcomes were accuracy (%) and reaction time (msec) for each of the three types of stimuli.

### Rapid Visual Information Processing (RVIP) task (focused/sustained attention)

The RVIP task requires the participant to monitor a continuous series of single digits for targets of three consecutive odd or even numbers. The white digits are presented on the black computer screen at the rate of 100 per minute for a total of three minutes; with eight correct target strings in each minute presented in pseudo-random order. The participant responded to the detection of a target string by pressing the response button as quickly as possible. In terms of task outcomes, RVIP is scored for accuracy (% detected), false alarms (number) and reaction time (msec). Given the generally poor performance on this task, a new outcome measure was derived taking into account the high rate of false alarms, which artificially inflated task accuracy. Response % accuracy was calculated by taking the number of correct responses and dividing this by the sum of the correct and incorrect responses and multiplying by 100 to give a percentage score.

### Stroop (selective attention, cognitive flexibility)

A series of 40 colour names (RED, YELLOW, GREEN, BLUE) were displayed on the screen one at a time. These were written in a coloured font. Participants were required to respond by pressing colour matched response box buttons based on the colour font the word was written in (not the colour the word depicts). Participants were shown a sequence of randomly ordered congruent (e.g. RED written in red font) or incongruent (e.g. RED written in blue font) stimuli. Outcomes were overall accuracy (% correct) and overall reaction time (msec) and the same individual outcomes for congruent stimuli and incongruent stimuli alone.

### Corsi Blocks Task (spatial working memory)

In this task nine identical blue squares appeared on screen in non-overlapping random positions. A set number of blocks changed colour from blue to red in a randomly generated sequence. The cursor was then unlocked, and participants repeated the sequence by clicking on the blocks using the mouse and cursor. The task was repeated five times at each level of difficulty, starting with 5 blocks, until the participant could no longer correctly recall the sequence, resulting in a span measure of nonverbal working memory, calculated by averaging the level of the last five correctly completed trials. For analysis, this span score was converted into a percentage accuracy score by taking the highest score achieved at any assessment by any participant (8.67) and converting this to 100%. Every other score was then adjusted to this highest score.

### Numeric Working Memory (working memory)

Five random digits from 1-9 were presented sequentially for the participant to hold in memory. This was followed by a series of 30 probe digits (15 targets and 15 distractors) for each of which the participant indicated whether it had been in the original series by a simple key press. The task consisted of 3 separate trials. Accuracy (% correct) and mean reaction time (msec) were recorded.

### Peg and Ball (executive function - planning)

Two configurations were shown on the screen. In each there was three coloured balls (blue, green, red) on one of 3 pegs. The configuration at the top of the screen is the goal configuration and participants must arrange the balls on the starting configuration (shown in the centre of the screen) to match the position of balls in the goal configuration. They must do this in the least number of moves possible. The number of errors recorded was converted to a % accuracy by taking the number of correct responses and dividing this by the sum of the correct and incorrect responses and multiplying by 100 to give a percentage score.

Summary of dependent variables

| **Measure** | **Outcomes** |
| --- | --- |
| Conners 3 Parent Report Short | inattention, hyperactivity, learning problems, defiance, family relations |
| Conners 3 Self Report Short | inattention, hyperactivity, learning problems, defiance, family relations |
| POMS | depression, anxiety, hostility, confusion, vigour, fatigue |
| PSS | Stress |
| VAMS | alertness, stress, tranquillity |
| STAI | Anxiety |
| Arrow flankers | overall % accuracy, overall RT, congruent % accuracy, congruent RT, incongruent % accuracy, incongruent RT, neutral % accuracy, neutral RT |
| Numeric working memory | % accuracy, RT |
| Stroop Task | overall % accuracy, overall RT, congruent % accuracy, congruent RT, incongruent % accuracy, incongruent RT |
| Corsi blocks | % accuracy |
| RVIP | % accuracy, RT, false alarms, % accuracy of response |
| Peg and ball | % accuracy |
| Speed of performance | numeric working memory RT + arrow flankers overall RT + Stroop task overall RT /3 |
| Accuracy of performance | arrow flankers overall accuracy + numeric working memory accuracy + Stroop task overall accuracy + Corsi blocks accuracy + RVIP accuracy of response + peg and ball accuracy /6 |
| Heart rate | beats per minute |
| Temperature | degrees Celsius |
| Blood pressure | systolic, diastolic mmHg |
| Heart rate variability | HRV triangular index |
