# Supplementary material for: Daily supplementation with lemon verbena extract decreases subjective energy and parental reports of hyperactivity in children displaying sub-clinical attention deficit hyperactivity disorder-type behaviours: A randomised controlled trial
Source: J Psychopharmacol. 2025 Apr 18;39(8):825–35. doi: 10.1177/02698811251324574 (PMC12287557; doi:10.1177/02698811251324574)
Supplement: sj-docx-2-jop-10.1177_02698811251324574 – Supplemental material for Daily supplementation with lemon verbena extract decreases subjective energy and parental reports of hyperactivity in children displaying sub-clinical attention deficit hyperactivity disorder-type behaviours: A randomised controlled [file sj-docx-2-jop-10.1177_02698811251324574.docx]

## Eligibility criteria

- In good health as reported by themselves and their parent/guardian
- Aged 8 to 17 years at the time of giving assent and parents giving consent.
- Have a sex and age-related BMI less than the 98^th^ centile according to the local NHS guidelines
- Rated by their parents as having a high score (T score of ≥60) on both the Conners 3-P(S) subscales of Inattention and Hyperactivity/Impulsivity
- Have no current diagnosis of ADHD
- Have no relevant food intolerances/ sensitivities/ allergies
- Are not currently using any illicit, herbal or recreational drugs including alcohol and nicotine products
- Are not currently taking prescription medications

*NOTE: the explicit exceptions to this are those taken ‘as needed’ in the treatment of hay fever, asthma, and medically prescribed vitamins*

- Have not taken dietary supplements e.g. vitamins, omega 3 fish oils etc. in the last 4 weeks
- Do not have a diagnosed neurological condition or learning/behavioural or neurodevelopmental difference (e.g. dyslexia, autism)
- Do not suffer from visual (including colour blindness) impairment that cannot be corrected with glasses or lenses (that may impact task performance in the opinion of the PI)
- Do not have any pre-existing diagnosed medical condition/illness which will impact taking part in the study

*NOTE: the explicit exception to this is controlled hay fever and asthma that requires infrequent medication*

- Consume less than 250 mg/day of caffeine
- Can complete all of the study assessments at the training visit
- Are not currently participating in other clinical or nutrition intervention studies, or have in the past 4 weeks
- Are compliant with regards to treatment consumption
- Have not taken antibiotics within the past 4 weeks
- Do not have any health condition that would prevent fulfilment of the study requirements (this includes non-diagnosed conditions for which no medication may be taken)
